# Notes and Notices

**Published:** 1890-12

**Authors:** 


### NOTES AND NOTICES.

REMOVALS.—Dr. Frances M. Morris, from Hotel Berkley to 157 Boylston Street, Boston, Mass. Dr. O. W. Lounsbury, from Cincinnati to Dayton, Ohio. Dr. L. H. Lemke, to Hixton, Jackson County, Wisconsin. He was erroneously reported, in our November number, as having gone to Higginsville, Mo. Dr. Charles H. Young, from Brooklyn to 319 West 44th Street, New York City. Dr. Hiram F. Smiley, from La Crosse, Wis., to South Englewood, Ill. Dr. G. Pompili, editor of *Revista Omiopatica*, from Chiavi d'ozo, to Via Cavour, 325, Rome, Italy.

Dr. Maria N. Johnson, who for a number of years has been located at 1732 Green Street, has, in consequence of the great increase in her practice, removed to 342 South Eighteenth Street, Philadelphia.

